# Demodicosis in two Holstein young calves

**DOI:** 10.1051/parasite/2011181089

**Published:** 2011-02-15

**Authors:** L. Martinelle, F. Dal Pozzo, B. Losson, P. Sarradin, C. Saegerman

**Affiliations:** 1 Department of Infectious and Parasitic Diseases, Faculty of Veterinary Medicine Boulevard de Colonster 20 B-4000 Liège Belgium; 2 INRA UE 1277, Experimental Infectiology Platform, INRA – Research Center of Tours Nouzilly France

**Keywords:** clinical epidemiology, demodicosis, *Demodex*, calves, emerging disease

Sir,

Demodicosis in cattle is caused by a microscopic mite, *Demodex bovis*. The parasites live sometimes in large numbers in the hair follicles and associated skin glands. The disease is well described and quite common in tropical zones, but rare and most likely underestimated in temperate regions, especially in Europe (Fisher, 1973; Matthes, 1994). Demodectic mange in cattle is known to be usually a chronic and benign disease. Lesions consist in papules and small nodules filled with a creamy-colored caseous material possibly associated with hair loss mainly observed in the periocular region, on the neck, and on the shoulders. Itching is usually absent. Under certain circumstances, such as stress, nutritional deficiencies, concurrent diseases and hot and humid weather the condition can extend to most parts of the body and lead to a loss of body condition.

**Fig 1.**
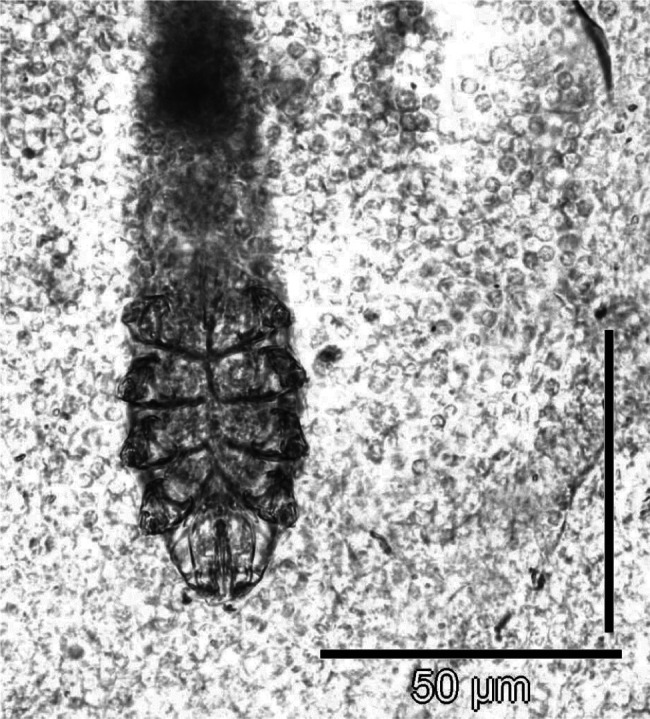
*Demodex bovis* is a worm-shaped, elongated mite (Acari: Prostigma), and that particular morphology makes its direct identification easy (photonic microscope ×200, after dilution in Phosphate Buffer Saline). Many parasites are found in the comedones embedded in the hair follicles.

**Fig 2.**
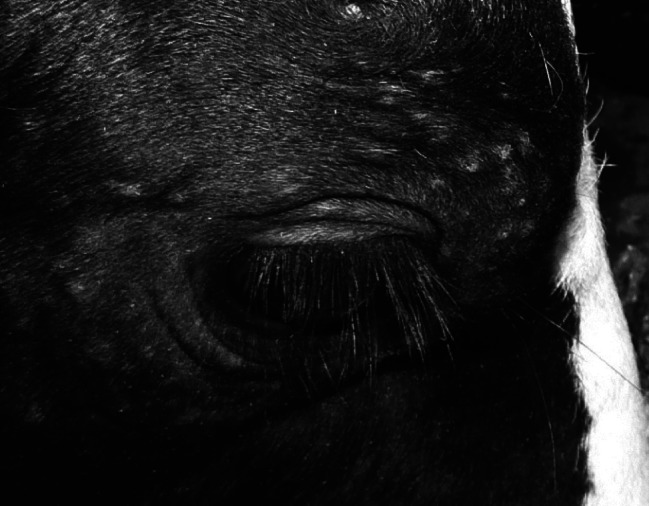
Many cutaneous nodes surround the eyes of the demodicidosis affected calves.

**Fig 3.**
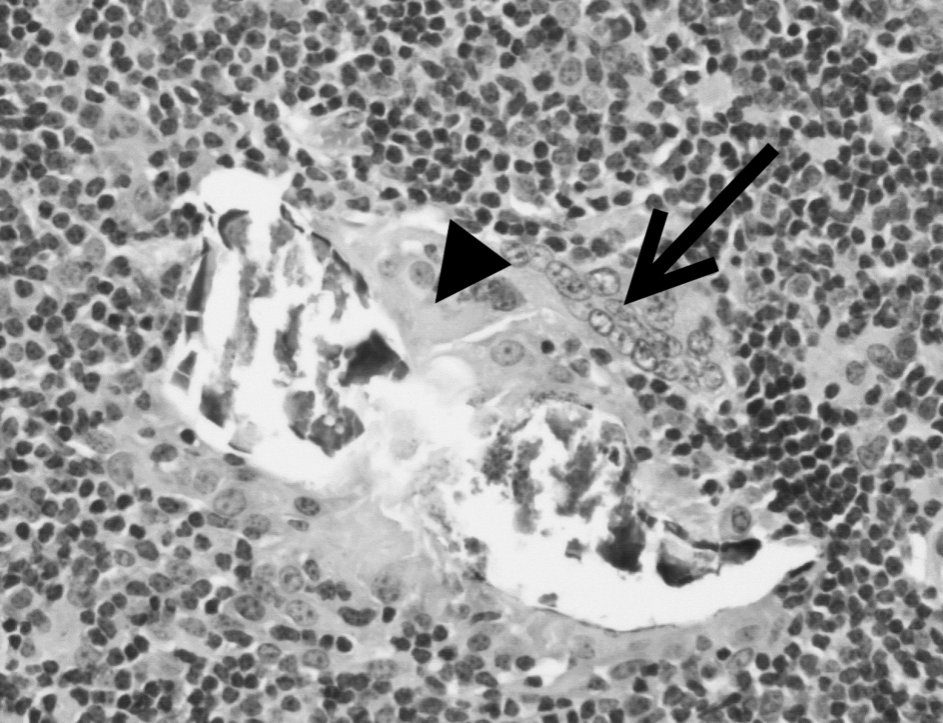
Around calcifying parasites fragments, macrophages (arrow head) and giant multinucleated cells (arrow) can be observed, typical of a granulomatous reaction. Surrounding this area, many lymphocytic cells frequently showing mitosis signs are visible (photonic microscope ×200).

We described a case of demodectic mange in two Holstein calves kept for experimental purpose. Clinical signs in conjunction with direct observation of the mites (Fig. 1) led to the demodicidosis diagnosis. The affection was incidentally detected and presented some unusual features regarding the classical epidemiological picture of the disease in temperate Europe. Demodicosis is considered to affect mainly young adult cattle, and the development of clinical signs is supposed to take between 3 to 6 months. Skin lesions, including papules surmounted with crusts and nodules with a diameter up to 2 cm were observed in calves aged 6 to 6.5 months (Fig. 2). Examination of slides made from a swollen parotidian lymph node revealed the presence of calcified *Demodex* (Fig. 3), most likely carried away by the blood or lymph flow once dead, which is in line with previous observations in the dog and cattle (Mbuthia *et al.*, 1994). To our knowledge no cases involving younger calves were described in European cattle to date.

*Demodex bovis* is usually considered to be transmitted from the dam to calf in the first weeks of life through nursing. The two affected calves were born in two distinct farms. At the time of the disease onset, these two animals were housed together. Two other groups of calves of the same age were kept in the same building but were physically separated from them. Evolution of the lesions followed a very similar pattern in both calves. The constitution of the experimental groups may have been responsible for a marked stress and a subsequent immunological impairment, which could allow the parasite to multiply. None of the other calves were affected. These elements point out the likely putative passage of *Demodex* from one calf to the other. Self cure was observed within 6 weeks. Demodectic mange is probably underdiagnosed in Europe. Its contribution to stress-related pathologies of calves should be investigated.
